# Intraoperative electrical stimulation promotes the short-term recovery of patients with cubital tunnel syndrome after surgery

**DOI:** 10.1186/s13018-023-03668-x

**Published:** 2023-04-03

**Authors:** Xuelei Zhang, Xiaolei Xiu, Ping Wang, Yaxin Han, Wenli Chang, Jianyong Zhao

**Affiliations:** 1 Department of Microsurgery, The Hebei Cangzhou Hospital of Integrated Traditional Chinese Medicine and Western Medicine, No. 31 Huanghe West Road, Cangzhou, 061000 Hebei China; 2 Hebei Key Laboratory of lntegrated Traditional and Western Medicine in Osteoarthrosis Research (Preparing), Cangzhou, China

**Keywords:** Intraoperative electrical stimulation, Ulnar nerve, Cubital tunnel syndrome

## Abstract

**Background:**

This study was designed to investigate whether intraoperative electrical nerve stimulation has effects on the short-term recovery of cubital tunnel syndrome patients after ulnar nerve release.

**Methods:**

Patients diagnosed as cubital tunnel syndrome were selected. At the same time, they received conventional surgery treatment. The patients were divided by a randomized digits table into two groups. The control group underwent conventional surgery, and the electrical stimulation (ES) group underwent intraoperative electrical stimulation. All the patients were tested for sensory and motor functions, grip strength, key pinch strength, motor conductivity velocity (MCV), and maximum compound muscle action potential (CMAP) before operation and 1 month and 6 months after operation.

**Results:**

In patients treated with intraoperative ES, the sensory and motor functions and the strength of muscle were significantly improved after 1-month and 6-month follow-up than the control group. After the follow-up, the patients in the ES group had significantly higher grip strength and key pinch strength than the control group. After the follow-up, the patients in the ES group had significantly higher MCV and CMAP than the control group.

**Conclusion:**

Intraoperative electrical stimulation of nerve muscle can significantly promote the short-term recovery of nerve and muscle functions after the surgery in cubital tunnel syndrome patients.

## Introduction

Cubital tunnel syndrome is a common neuropathy [1]. Cubital tunnel is lined with the ulnar nerve and relative vessels [2]. The ulnar nerve is a multifascicular trunk and the roof of the cubital tunnel showed the presence of superimposed layers [3]. The main cause of cubital tunnel syndrome is the enduring, repetitive flexion of elbow that raises the pressure inside the cubital tunnel and strains the ulnar nerve, affecting the microcirculation within the nerve and causing ischemia and hypoxia resulting in nerve damage [4]. Chronic compression leads to demyelination and the distortion of axonal structures, followed by undesirable remyelination [5]. The motor symptoms include dyspraxia and weakness of the hand and the sensory symptoms include pain, hypoesthesia or anesthesia, and cutaneous dysesthesias [5]. For patients with mild symptoms, they can heal spontaneously without surgery [6]. Early cubital tunnel syndrome can be treated conservatively, but cases with more than 3 months often require surgical release, such as endoscopic cubital tunnel release [7, 8].

Electrical stimulation is attracting increasing attention due to its promising role in promoting neuromuscular movement and facilitating peripheral nerve regeneration [9]. Numerous studies have demonstrated that electrical stimulation of the muscles innervated by injured peripheral nerves can promote nerve function recovery [10]. Electrical stimulation promotes peripheral nerve axon regeneration may by the upregulation of intracellular cyclic adenosine monophosphate (cAMP) levels, which causes enhanced neurotrophic factor expression [11]. Neurotrophins such as neurotrophin-4 (NT-4) and brain-derived neurotrophic factor (BDNF) contribute to nerve recovery [11]. In both animal and human studies, electrical stimulation can enhance axonal regeneration after the surgery [12]. Another research has demonstrated that intraoperative electrical stimulation improves the scores of Disabilities of the Arm, Shoulder, and Hand (DASH) questionnaire in severe cubital tunnel syndrome patients [13]. Herein, this research aimed to explore whether intraoperative electrical stimulation has beneficial effects on the short-term recovery of cubital tunnel syndrome patients.

## Methods

### Participants

Participants in this randomized clinical trial were cubital tunnel syndrome patients who required surgical treatment after ineffective conservative treatment at our hospital from June 2018 to June 2021. The patients were divided by a randomized digits table into two groups. The control group underwent conventional surgery, and the electrical stimulation (ES) group underwent intraoperative electrical stimulation. The study was approved by the ethics committee of the Hebei Cangzhou Hospital of Integrated Traditional Chinese Medicine and Western Medicine, and all the participants signed informed written consent.

The patients were diagnosed with cubital tunnel syndrome through physical examination, electromyography, and ultrasonography, and the conservative treatment was ineffective. The preoperative physical examination met all the criteria: 1. numbness of ulnar nerve innervation region; 2. dorsal interosseous muscle atrophy; 3. clinical Tinel's sign or positive for the elbow flexion test; 4. clawhand deformity; 5. fractioned measurement of the nerve conduction velocity with stimulation of the ulnar nerve at the wrist, distally and proximally to the cubital tunnel (with a distance of more than 10 cm).

The inclusion criteria were: 1. diagnosed with cubital tunnel syndrome; 2. ineffective conservative treatment; 3. having paresthesia, numbness, muscle weakness, or pain in ulnar nerve distribution; 4. having positive electromyography and Tinel's nerve percussion test results.

The exclusion criteria were: 1. having previous elbow injury or other secondary associated pathologies; 2. having previous peripheral neuropathy; 3. having metallic implants or non-magnetic resonance imaging (MRI) safe active implants; 4. having diabetes mellitus, vascular disease, or other polyneuropathies.

**Fig. 1:**
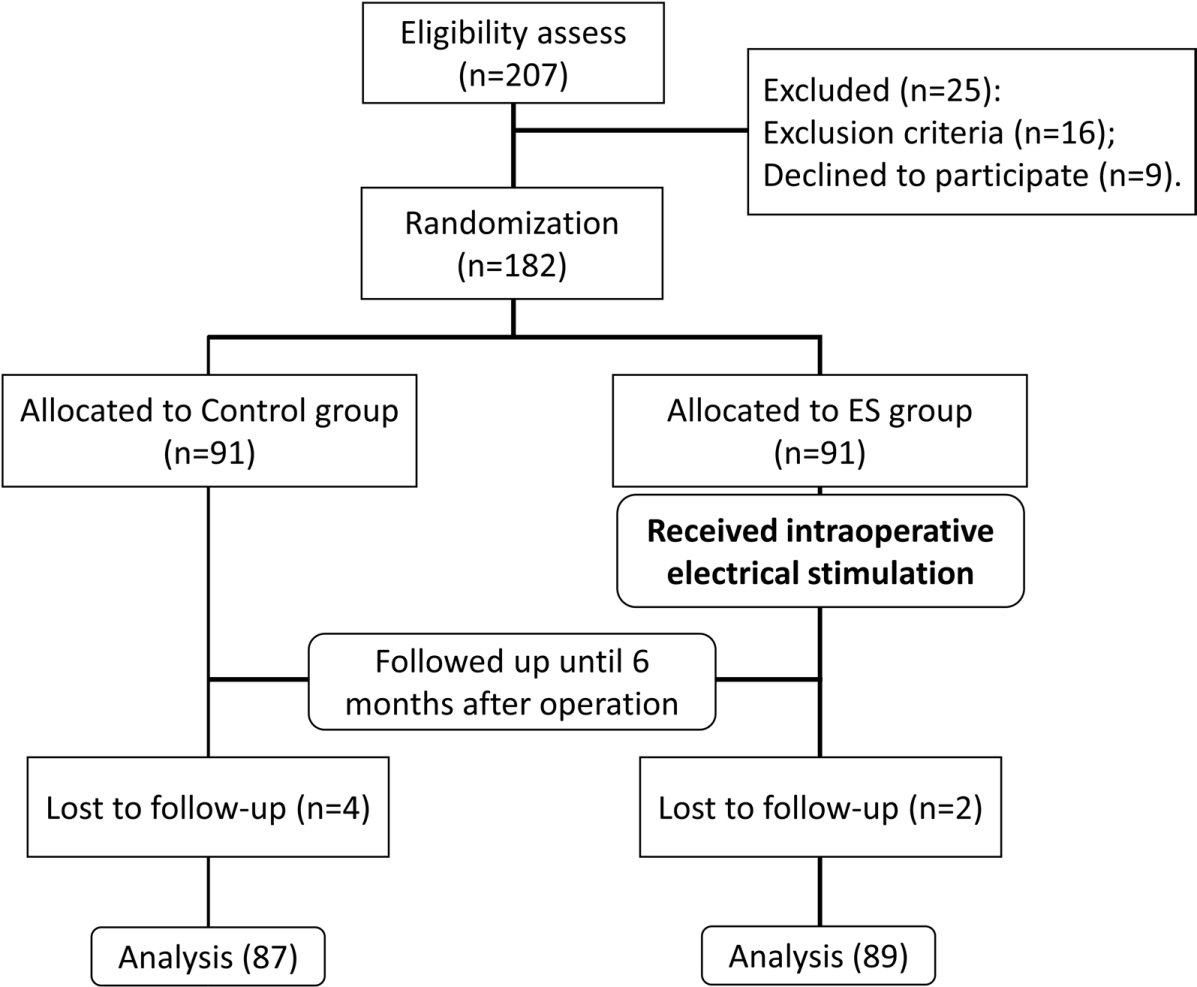
Study flow diagram

Of the 207 patients included in the eligibility assess, 25 were excluded. A total of 182 patients were randomized into the two groups. Participants were followed up for 6 months. Finally, 87 cases in the control group and 89 cases in the ES group were analyzed. Study flow diagram was shown in Fig. 1. Researchers who conduct detection and data collection and analysis were blind to the grouping.

### Surgical procedures

All surgeries were performed by the same surgical team and under general anesthesia. The ulnar nerve was released at all common sites of compression around the elbow. An in-situ decompression was performed in the majority of cases. Intraoperative electrophysiological monitoring was performed before and after the decompression. The compound muscle action potentials (CMAP) amplitudes of the abductor digiti minimi (ADM) muscle and the motor nerve conduction velocity (MNCV) in the ulnar nerve were recorded. After the decompression, patients in the ES group received electrical stimulation. In this study, alternative high- and low-frequency electrical stimulation was used (100 mA, 2 Hz/15 Hz, 15 min). After the decompression, patients in the ES group received electrical stimulation on the most severely compressed site. In this study, alternative high- and low-frequency electrical stimulation was used (100 mA, 2 Hz/15 Hz, 15 min). Electrical stimulation was performed by Grass SD9 stimulator (Grass Technologies, Warwick, Rhode Island). The proximal wire electrode was connected to the cathode and the distal to the anode.

The conservative treatments included oral analgesics, nonsteroidal anti-inflammatory drugs, and neurotrophic drugs; in addition, the patient has a history of treatment related to acupoint application of traditional Chinese medicine for promoting blood circulation and reducing swelling.

### Outcome assessment

Motor and sensory functions were evaluated according to the British Medical Research Council (BMRC) scale. Excellent: Complete recovery of muscle strength on the affected side and complete recovery of superficial pain and tactile sensation. Good: Incomplete recovery of muscle strength on the affected side and incomplete recovery of superficial pain and tactile sensation. Muscle contraction can still move joint against week resistance. Fair: Incomplete recovery of muscle strength on the affected side. Partial recovery of superficial pain and tactile sensation. Muscle strength is further reduced. Poor: No recovery of muscle strength on the affected side and superficial pain and tactile sensation.

The strength of muscle was assessed by the Lovett scale. V: Muscle contracts normally against full resistance. IV: Muscle strength is reduced but muscle contraction can still move joint against resistance. III: Muscle strength is further reduced such that the joint can be moved only against gravity with the examiner's resistance completely removed. II: Muscle can move only if the resistance of gravity is removed. I: Fasciculations are observed in the muscle or only a trace or flicker of movement is seen or felt in the muscle.

Key pinch strength was evaluated by pinch gauge (B&L Engineering, Santa Ana, California). Grip strength was evaluated by Jamar dynamometer (Sammons Preston Rolyan, Bolingbrook, Illinois).

### Electromyography

Electromyography examination was performed by a standard EMG system (Nicolet Synergy, Natus Medical Incorporated, San Carlos, USA) through the most widely used methods with good reliability [12]. The positive pole was placed 6 cm above the elbow on the projection of the cubital tunnel of the affected limb, and the negative pole was placed on the ulnar side of the hand at the ADM muscle. MCV and the maximum CMAP were recorded.

### Statistical analysis

SPSS 17.0 software (SPSS Inc., Chicago, IL, USA) was used for statistical analysis. Values were shown as n (percentage, %) or mean ± SD. The differences between each group were derived from Mann–Whitney test or Two-way ANOVA followed by Tukey's multiple comparisons test. Fisher’ s exact test or Chi-square test was used for assessing distribution of observations between two groups. *p* value < 0.05 was considered statistically significant.

## Results

Table 1 showed the baseline characteristics of patients. The age, body mass index (BMI), gender, the site of disease, and the duration of symptoms in the two groups had no significant difference (all *p* > 0.05).

**Table 1:** Baseline characteristics of cubital tunnel syndrome patients treated with intraoperative electrical stimulation (ES) and control

Characteristics	Study groups		*p* value
	Control (*n* = 87)	ES (*n* = 89)	
Age (years)	52.35 ± 8.94	53.13 ± 9.07	0.184
BMI (kg/m^2^)	22.58 ± 3.59	22.27 ± 3.91	0.208
Gender			
Male	51 (58.6%)	48 (53.9%)	0.547
Female	36 (41.4%)	41 (46.1%)	
Cubital tunnel syndrome side			
Left	19 (21.8%)	15 (16.8%)	0.444
Right	63 (72.4%)	65 (73.1%)	
Both	5 (5.8%)	9 (10.1%)	
Duration of symptoms (months)	16.85 ± 5.26	17.12 ± 6.07	0.227

Values were shown as *n* (percentage, %) or mean ± SD. *p* values for each group were derived from Mann–Whitney test. Fisher’s exact test or Chi-square test was used for assessing distribution of observations between two groups

The sensory and motor functions of cubital tunnel syndrome patients after 1-month and 6-month follow-up were evaluated by the BMRC scale. As shown in Table 2, in patients treated with intraoperative ES, the sensory and motor functions were significantly improved after 1-month and 6-month follow-up than the control group.

**Table 2:** Comparison of sensory and motor functions of cubital tunnel syndrome patients treated with intraoperative electrical stimulation (ES) and control

	Study groups		*p* value
	Control (*n* = 87)	ES (*n* = 89)	
1 months after the operation			
Excellent	6 (6.9%)	15 (16.9%)	< 0.001
Good	25 (28.7%)	46 (51.7%)	
Fair	36 (41.4%)	16 (17.9%)	
Poor	20 (23.0%)	12 (13.5%)	
6 months after the operation			
Excellent	21 (24.1%)	32 (35.9%)	0.029
Good	33 (37.9%)	41 (46.1%)	
Fair	26 (29.9%)	13 (14.6%)	
Poor	7 (8.1%)	3 (3.4%)	

Values were expressed as *n* (percentage, %). *p* value was derived from Chi-square test

The strength of muscle was assessed by the Lovett scale. As shown in Table 3, in patients treated with intraoperative ES, the strength of muscle was significantly enhanced after 1-month and 6-month follow-up than the control group.

**Table 3:** Comparison of Lovett muscle grading of cubital tunnel syndrome patients treated with intraoperative electrical stimulation (ES) and control

	Study groups		*p* value
	Control (*n* = 87)	ES (*n* = 89)	
1 months after the operation			
I	6 (6.9%)	2 (2.2%)	0.008
II	23 (26.4%)	14 (15.7%)	
III	30 (34.5%)	20 (22.5%)	
IV	20 (23.0%)	37 (41.6%)	
V	8 (9.2%)	16 (18.0%)	
6 months after the operation			
I	0 (0%)	0 (0%)	0.048
II	15 (17.3%)	9 (10.1%)	
III	31 (35.6%)	21 (23.6%)	
IV	20 (23.0%)	22 (24.7%)	
V	21 (24.1%)	37 (41.6%)	

Values were expressed as *n* (percentage, %). *p* value was derived from Chi-square test

**Fig. 2:**
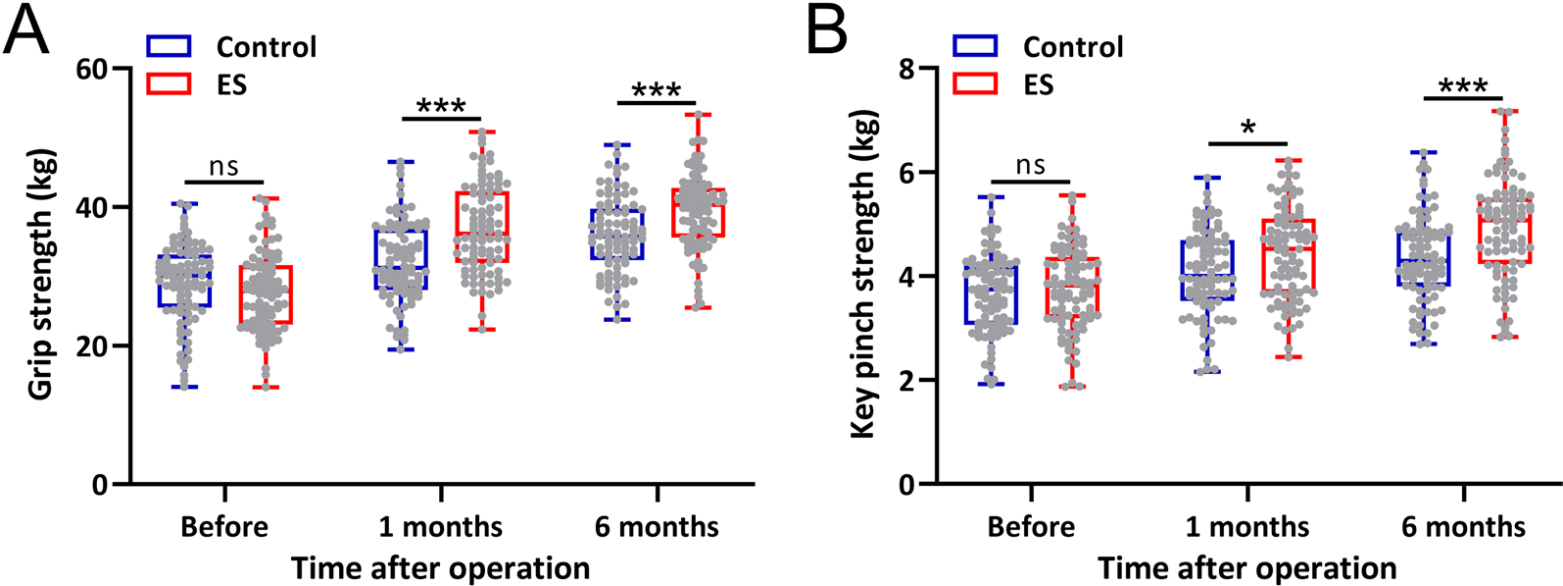
Comparisons of Grip strength (**A**) and Key pinch strength (**B**) before the surgery and 1 month, 6 months after the surgery between cubital tunnel syndrome patients treated with intraoperative electrical stimulation (ES) and control. Box plot was used to present the data. *p < 0.05, ***p < 0.001 and ns means no significance. Two-way ANOVA followed by Tukey's multiple comparisons tests

The key pinch strength and grip strength of the cubital tunnel syndrome patients were also evaluated. Before the surgery, both key pinch strength and grip strength showed no significant differences between the two groups (Fig. 2A and B). At 1 month and 6 months after the surgery, the key pinch strength and grip strength in the ES group were significantly higher than the control group (Fig. 2A and B).

**Fig. 3:**
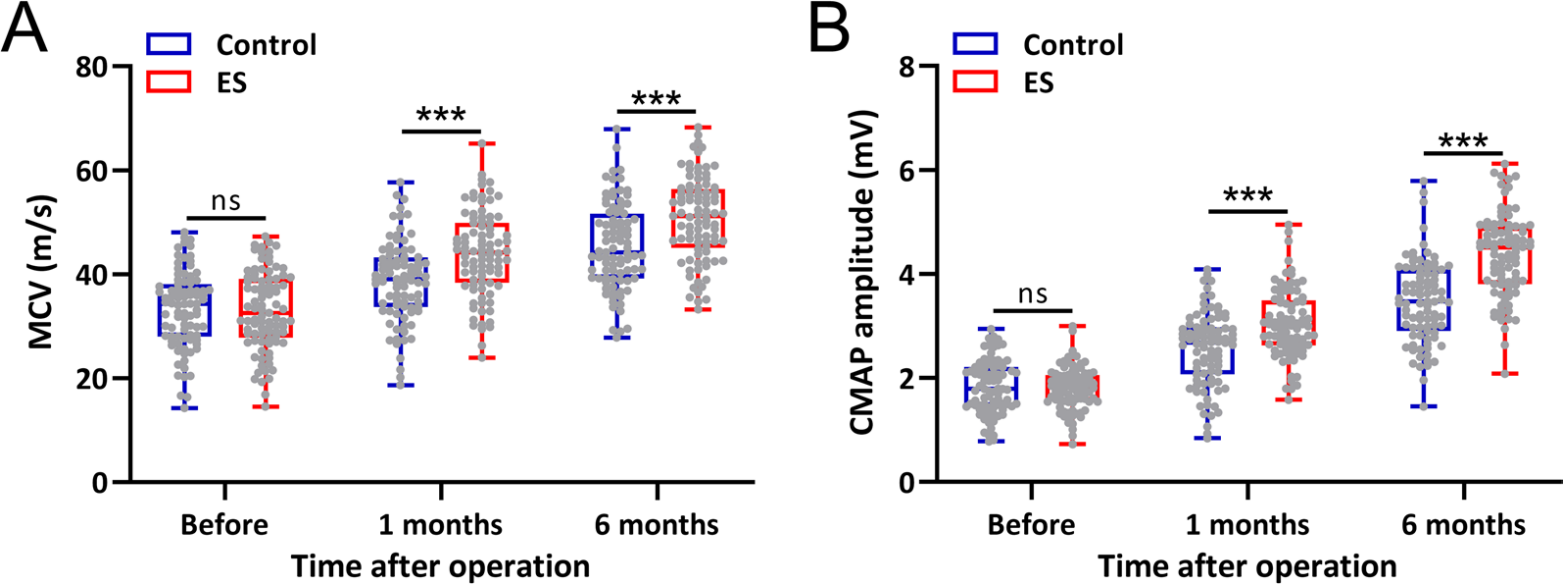
Comparisons of motor conductivity velocity (MCV) (**A**) and maximum compound muscle action potential (CMAP) (**B**) before the surgery and 1 month, 6 months after the surgery between cubital tunnel syndrome patients treated with intraoperative electrical stimulation (ES) and control. Box plot was used to present the data. ***p < 0.001 and ns means no significance. Two-way ANOVA followed by Tukey's multiple comparisons tests

Before the surgery, both MCV and CMAP showed no significant differences (Fig. 3A and 3B). In the ES group, the MCV and CMAP of the patients were significantly higher than the control group at 1 month and 6 months after the surgery (Fig. 3A and B).

## Discussion

A large number of researchers have carried out studies related to the repair function of electrical stimulation in nerve injury, proving effectiveness and reliability from a practical point of view [14]. The relevance of adding some electrical stimulation to the process of regeneration of nerve axons has also been proved within rat model [15, 16]. The application of electrical stimulation has a positive effect on the recovery of muscle nerve damage [17]. The application of electrical stimulation can effectively delay muscle atrophy caused by nerve loss. The corresponding mechanisms are focused on the following aspects: 1. regulation of the target tissue metabolism after denervation, which accelerates blood circulation in the muscle; 2. delaying the degenerative changes in muscle proteins and reducing the loss of muscle glycogen; 3. delaying the process of denervation atrophy and reducing the ATP level; 4. increasing the efficiency of glycogen protein synthesis and increasing the total amount of non-collagenous proteins.

Despite the potential of damaged peripheral nerves to regenerate axons, functional recovery after severe nerve injury in humans remains poor [18]. When axons regenerate, the regenerated nerve fibers are unable to locate the correct innervation target and misorientation is detrimental to nerve repair, which can lead to uncoordinated movements [19]. Koppes has demonstrated that the speed and success of nerve repair can be improved by directly promoting axonal growth and directional effects after the application of appropriate electrical stimulation, as a way to increase the release of neurogenic factors 11-fold [20]. Gordon suggested that in the process of nerve transection and surgical repair, some electrical stimulation for the denervated muscle has a positive impact and can effectively enhance the rate of nerve regeneration in the muscle itself [11, 21]. The promotion of nerve recovery by electrical stimulation includes: (i) promotion of nerve growth factor expression and its neurotropic effect; (ii) current effect: under the effect of positive and negative electric field, the positively charged nerve growth factor starts to move gradually toward the negative electrode (distal to the nerve injury), and this condition can effectively accelerate the regeneration of the nerve itself; (iii) reduction of calcium ion levels, which has a positive effect on improving the blood flow inside the injured nerve.

In this study, we applied an electromyograph to intervene in the postoperative recovery of patients with cubital tunnel syndrome in the form of electrical stimulation, and two tests were applied to assess the patient's neurological recovery, i.e., electromyographic indices, and sensory and motor functions to assess the patient's clinical recovery. The electromyograph allows the adjustment of stimulation parameters and treatment time, as well as the postoperative assessment of all electrophysiological aspects of the muscle, thus providing better objectivity.

In this study, sensory and motor functions were evaluated at 1 and 6 months of postoperative follow-up. The ES group showed a significantly faster motor and sensory function recovery compared to the control group. The performance of intraoperative electrical stimulation enhanced motor and sensory function recovery in cubital tunnel syndrome patients.

The Lovett muscle strength grading was used to evaluate the finger muscle strength of the patients at 1 and 6 months postoperative follow-up. The comparison showed that the recovery of muscle strength in the ES group was also superior to the control group. The performance of intraoperative electrical stimulation enhanced the recovery of muscle strength after surgery.

The key pinch strength and grip strength were compared between the control group and the ES group before surgery and at the postoperative follow-up of 1 and 6 months. There was no significant difference between the two groups before surgery, but the grip strength and key pinch strength recovered faster after surgery in the ES group.

The CMAP and the MCV of the ulnar nerve were compared at 1 and 6 months of postoperative follow-up. The MCV and CMAP were not significantly different between the two groups before surgery, but the postoperative recovery was significantly better in the ES group than in the control group.

Due to the small sample size of this experiment and the fact that only cubital tunnel syndrome after ineffective conservative treatment was selected as a therapeutic target for nerve injury, while other peripheral nerve injuries (e.g., peripheral nerve dissection due to upper limb trauma, carpal tunnel syndrome due to long-term entrapment) have not been further studied, we will next expand the sample size and further investigate the mechanism of electrical stimulation for nerve recovery.

## Conclusion

In conclusion, intraoperative electrical stimulation of nerve muscle can significantly promote the short-term recovery of nerve and muscle functions after the surgery in patients with cubital tunnel syndrome.

## Data Availability

The data that support the findings of this study are available from the corresponding author, Lei Chen, upon reasonable request.
